# Advances in drug addiction research using *Caenorhabditis elegans*: behavioral and molecular mechanisms

**DOI:** 10.3389/fcell.2026.1880079

**Published:** 2026-07-01

**Authors:** Lihua Yang, Shuang Wu, Jiale Liu, Wenjun Wang, Qinhong Yin, Elizabeth Rosalind Thomas, Xiang Li

**Affiliations:** 1 Yunnan Police College, Kunming, China; 2 Department of Biochemistry and Molecular Biology, School of Basic Medical Sciences, Southwest Medical University, Luzhou, China; 3 Department of Medical Microbiology, PGIMER, Chandigarh, India; 4 Health Science Center, Xi’an Jiaotong University, Xi’an, China

**Keywords:** behavioral changes, *Caenorhabditis elegans*, drug addiction, neuroplasticity, signaling

## Abstract

Drug addiction is a complex, chronic, and relapsing neurological disorder characterized by persistent neuroadaptation and a substantial public health burden. Because of its simple nervous system, genetic tractability, short life cycle, transparent body, and quantifiable behavioral phenotypes, *C. elegans* (*Caenorhabditis elegans*) has become a useful complementary model for studying selected aspects of drug-induced behavioral adaptation. This review summarizes recent advances in the use of *C. elegans* to study opioids, amphetamine-type stimulants, cocaine, ketamine, ethanol, nicotine, and related anesthetic or depressant-type compounds. We discuss commonly used behavioral paradigms, including conditioned cue preference, swimming-induced paralysis, tolerance assays, withdrawal-like responses, chemotaxis, and locomotor adaptation, together with dopaminergic, cholinergic, serotonergic, gamma-aminobutyric acid (GABA)-mediated, neuropeptidergic, ion-channel, oxidative-stress, transcriptional, and epigenetic mechanisms. The main limitations of this model are also considered, including the lack of mammalian reward-circuit complexity, nematode-specific pharmacokinetic features, cuticle permeability, and limited direct translational validation. Overall, *C. elegans* is best used as a mechanistic and screening-level model to identify conserved pathways and candidate targets that require further validation in mammalian systems.

## 1 Introduction

Drug addiction is a complex, chronic, and relapsing disorder. It is also an important public health problem worldwide. The World Drug Report 2025 reported that about 316 million people used drugs in 2023, accounting for nearly 6% of people aged 15–64 years (United Nations Office on Drugs and Crime, 2025). These data show that addiction research still needs tractable experimental models to examine drug-induced behavioral and molecular changes. Mammalian models are necessary for studies related to clinical translation. However, *Caenorhabditis elegans* provides a useful complementary model for studying early drug-induced behavioral adaptation, conserved signaling pathways, and candidate molecular targets.

Drug addiction is regarded as a chronic disorder of the brain reward system, in which abnormal dopamine transmission and neural circuit remodeling are closely associated with addictive behaviors. A growing body of evidence indicates that the mechanisms underlying drug addiction are highly complex and involve genetic susceptibility, environmental influences, and the effects of prolonged drug exposure on the brain. These factors contribute to compulsive drug use and relapse mainly through regulation of the dopaminergic reward pathway, the hypothalamic-pituitary-adrenal (HPA) axis, synaptic plasticity, and transcriptional and epigenetic remodeling (Volkow and Blanco, 2023). Nonetheless, the mammalian nervous system is highly complex. In particular, dopaminergic circuits are composed of large numbers of neurons, which increases the difficulty of dissecting the underlying mechanisms at the single-cell and fine-circuit levels. In this context, *C. elegans* offers experimental advantages including a compact and well-defined nervous system, powerful genetic tractability, and conservation of key signaling pathways, making it a useful complementary model for mechanistic studies (Torres et al., 2025). At present, support for *C. elegans* as a model for addiction research mainly comes from behavioral studies, genetic manipulation, and pharmacological investigations, rather than from clinically validated translational systems (Schafer, 2004). Notably, nematodes have been shown to display addiction-related behavioral phenotypes, including conditioned cue preference, preference-based responses to drug-related stimuli, and tolerance, and these phenotypes are closely linked to conserved neurotransmitter signaling pathways such as dopamine signaling (Ide and Ikeda, 2024).

Neural adaptations associated with addiction may be driven by repeated drug exposure, changes in synaptic plasticity, and dysregulation of transcriptional and epigenetic mechanisms (Nestler and Lüscher, 2019). Importantly, addiction-related neurobiological changes may emerge before overt compulsive drug use becomes established (Everitt et al., 2008). Continuous neural adaptation and circuit remodeling are important features of drug-induced neuroplasticity and may occur before stable addiction-like behavioral phenotypes become established (Lüscher and Malenka, 2011). Thus, drug-induced neuroadaptations may initially represent responses to exogenous stimuli but later contribute to the maintenance of addiction and relapse (Volkow et al., 2019). In nematodes, sustained drug exposure can induce conditioned preference, tolerance, and withdrawal-like responses, suggesting that they can serve as an important model for studying early adaptation to addiction and later pathological transformation. Characterizing drug-induced behavioral plasticity and associated neurotransmitter signaling in *C. elegans* may help clarify addiction mechanisms and identify potential intervention targets. Therefore, by combining a fully mapped nervous system, conserved neurotransmitter pathways, powerful genetic tractability, and rapidly quantifiable behavioral outputs, *C. elegans* provides a tractable platform for linking drug-induced neural plasticity to addiction-related behavioral phenotypes and for identifying potential intervention targets.

This review integrates current evidence on drug-induced behaviors and molecular adaptations in *C. elegans*. First, we summarize the behavioral phenotypes and potential molecular mechanisms induced by different addictive drugs in nematodes. Second, we discuss the value of the nematode model in elucidating conserved neurobiological mechanisms and screening potential intervention targets. Third, we identify key gaps in current research to inform future mechanistic studies and translational applications.

## 2 Advantages and biological basis of the nematode model in addiction research

*C. elegans* is a widely used invertebrate model organism in neurobiology. It has a fully characterized map of neurons and synapses. A large number of mutant strains are available. Powerful genetic and molecular tools allow precise manipulation of its genome and gene expression (Randi et al., 2023; Lucio et al., 2018). *C. elegans* was the first multicellular organism with a fully sequenced genome and harbors approximately 19,000 protein-coding genes (Hillier et al., 2005). Notably, over 65% of its genes have homologs associated with human diseases (C. elegans Sequencing Consortium, 1998). In addition to its genetic tractability, *C. elegans* has stereotyped internal anatomy that includes a muscular pharynx, intestine, excretory system, gonad, and cuticle-based body surface (Sundaram and Buechner, 2016). Its nervous system is highly defined, with 302 neurons in adult hermaphrodites that are organized into sensory neurons, interneurons, and motor neurons connected through chemical synapses and electrical gap junctions (Yu et al., 2022; Emmons, 2024). Despite this simplicity, *C. elegans* contains conserved neurotransmitter systems, including dopaminergic, serotonergic, cholinergic, GABAergic, and glutamatergic signaling, which regulate major behaviors relevant to drug responses (Chase and Koelle, 2007; McDonald et al., 2006). The identity, developmental lineage, and synaptic connectivity of each neuron are completely mapped (Witvliet et al., 2021). *C. elegans* displays diverse and quantifiable behaviors, including egg-laying, defecation, feeding, and locomotion (Liu X. et al., 2025). By combining behavioral assays with genetic and molecular approaches, target genes and signaling systems mediating drug responses have been identified in *C. elegans*, including dopaminergic and other conserved neurotransmitter pathways (Giunti et al., 2021).

**FIGURE 1:**
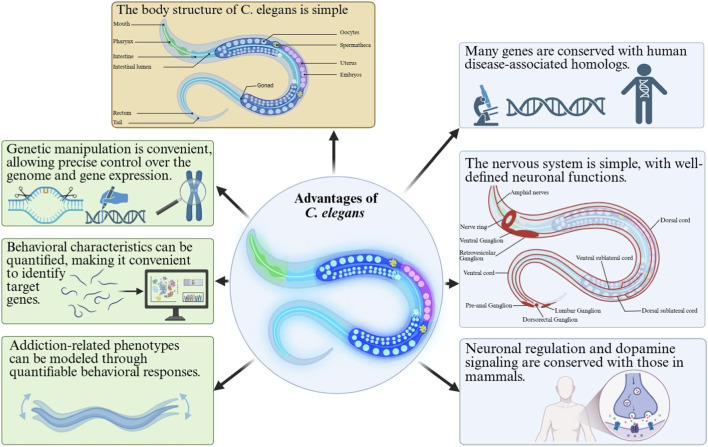
Advantages and biological basis of *Caenorhabditis elegans* (*C. elegans*) in drug addiction research. The schematic shows major visceral organs, the simple nervous system, and key advantages of *C. elegans*, including genetic tractability, quantifiable behavioral phenotypes, conserved disease-associated homologs, and conserved neuronal regulation.

*C. elegans* possesses a conserved monoaminergic system with signaling properties that are highly similar to those of vertebrates, including humans (McDonald et al., 2006). Dopamine regulates multiple functions in *C. elegans*, such as learning, locomotion, egg-laying, defecation, tactile habituation, food sensing, and mating behaviors (Pribadi et al., 2023). Beyond dopamine signaling, *C. elegans* contains conserved serotonergic, cholinergic, GABAergic, glutamatergic, and neuropeptidergic systems that regulate locomotion, feeding, learning, egg-laying, sensory processing, and behavioral adaptation. These pathways are relevant to different classes of addictive substances: cholinergic signaling is central to nicotine responses, serotonergic signaling contributes to cocaine-induced locomotor changes, GABAergic and ion-channel pathways are implicated in alcohol responses, and neuropeptide-G protein-coupled receptor (GPCR) signaling participates in opioid-like and ethanol-related behaviors. Hermaphrodites contain eight dopaminergic neurons. Males possess an additional six pairs of dopaminergic neurons involved in mating behavior. These neurons synthesize dopamine and express the dopamine transporter (DAT-1), which mediates dopamine reuptake from the synaptic cleft. In *C. elegans*, the *cat-2* gene encodes tyrosine hydroxylase, the rate-limiting enzyme in dopamine synthesis, required for drug-induced behavioral responses. Its role is consistent with the involvement of dopaminergic neurotransmission in human addiction. Drug-exposed *C. elegans* also exhibit addiction-related features, including sensitization, cross-sensitization, tolerance, and cross-tolerance. Overall, *C. elegans* can model selected aspects of drug-induced behavioral adaptation and provides a platform for identifying neural systems and molecular mechanisms underlying drug effects (Giunti et al., 2021) (Figure 1).

## 3 Behavioral paradigms for addiction-related phenotypes

### 3.1 Conditioned place preference model

The conditioned place preference (CPP) assay is a classical Pavlovian paradigm used to assess the rewarding or aversive effects associated with drug exposure (McKendrick and Graziane, 2020). In mammalian studies, CPP is based on the association between the pharmacological effects of a drug and a specific environmental context. In *C. elegans*, this paradigm is usually adapted as conditioned chemosensory-cue preference (CCP), in which a neutral sensory cue, such as a salt, odorant, or food-related cue, is paired with drug exposure during conditioning (Kim et al., 2024). During the subsequent drug-free test phase, increased approach to the drug-paired cue is interpreted as conditioned preference, whereas reduced approach may indicate aversive learning (Cho et al., 2016). In invertebrates, reinforcement-related behaviors are strongly influenced by biogenic amine signaling. Because these pathways are evolutionarily conserved, they provide a useful framework for studying conserved mechanisms of addiction (Rosikon et al., 2023). The biogenic amine pathways are functionally conserved in the nematode *C. elegans*. This model has been used to examine addiction-related associative learning in nematodes. Cocaine and methamphetamine can induce conditioned preference for drug-paired salt or food cues, and this response is reduced in dopamine-deficient mutants, indicating that dopaminergic signaling is required for stimulant-associated cue conditioning (Engleman et al., 2018). Opioid-like signaling involving neuropeptide receptor 17 (NPR-17) has also been implicated in morphine- or opiate-related behavioral regulation in *C. elegans* (Cheong et al., 2015; Mills et al., 2016). More recently, nicotine CCP has been used to evaluate nicotine preference and seeking, with evidence supporting the involvement of nicotinic acetylcholine receptors and dopamine signaling (Sellings et al., 2013). In this context, *C. elegans* provides a tractable model for analyzing molecular and genetic responses to addictive substances and for screening candidate modulators (Li et al., 2025). Therefore, CCP provides a useful behavioral framework for linking drug-associated cue learning with conserved neurotransmitter pathways in *C. elegans* (Rosikon et al., 2023). However, because the nematode assay relies on chemosensory cues rather than complex spatial contexts, it should be interpreted as a simplified model of drug-associated preference rather than a direct equivalent of mammalian CPP (Caldwell, 2023).

### 3.2 Swimming-induced paralysis model

Under normal conditions, *C. elegans* exhibits sinusoidal crawling on solid surfaces, but its locomotion transitions into a vigorous “thrashing” motion in liquid environments. Mutants lacking the dopamine transporter DAT-1 maintain normal crawling on solid surfaces but develop paralysis in liquid, a phenotype known as swimming-induced paralysis (SWIP) (Refai et al., 2022). This paralysis can also be induced pharmacologically by blocking DAT-1, which leads to increased synaptic dopamine levels (Kudumala et al., 2019). SWIP is thought to result from impaired dopamine reuptake, which increases extracellular dopamine levels and disrupts motor activity. Accordingly, the SWIP paradigm has been widely used to assess abnormalities in dopamine signaling. The intensity of SWIP is tightly governed by the accumulation of extracellular dopamine and its subsequent regulation of downstream receptors. At the presynaptic interface, the dopamine transporter does more than just recycle dopamine from the synaptic cleft; it can also depolarize neurons through a unique channel-like mechanism. This property may help explain how altered transporter kinetics influence neural activity and contribute to addiction-related behavioral phenotypes (Carvelli et al., 2004).

At the transcriptional level, *rnt-1*, a Runx-family transcription factor, acts together with its cofactor BRO-1 to regulate gene expression programs relevant to this phenotype. Robinson et al. (2019) showed that loss of *rnt-1* function induces SWIP, likely through altered interactions between dopaminergic neurons and body wall muscles (Robinson et al., 2019). Furthermore, recent evidence suggests that cannabidiol (CBD) and cannabidivarin (CBDV) induce SWIP-like phenotypes. These effects are abolished when the *dop-3* receptor is mutated, suggesting that CBD and CBDV may act through DOP-3-dependent mechanisms *in vivo* (Shrader et al., 2020). Overall, the *C. elegans* SWIP model may be useful for assessing dopaminergic dysfunction and for screening the behavioral effects of novel psychostimulants.

### 3.3 Tolerance assays

Drug tolerance refers to a reduced sensitivity to an addictive substance following repeated exposure, necessitating higher doses to achieve the same effect. This phenomenon is common in opioid, stimulant, and alcohol addiction. The development of tolerance mainly involves neuroadaptive processes that reduce brain sensitivity to reward signals. In addiction research, tolerance is usually interpreted as an adaptive neurobiological process that reflects altered sensitivity of neural circuits to repeated or prolonged drug stimulation (Nestler and Lüscher, 2019). In *C. elegans*, tolerance can be quantified using rapidly measurable behavioral readouts, particularly locomotion-based assays (Katner et al., 2019). For ethanol, acute functional tolerance is commonly evaluated by comparing the degree of locomotor impairment during early exposure with partial behavioral recovery during continued exposure (Jee and Batsaikhan, 2024). This phenotype suggests that worms can adapt to the intoxicating effects of ethanol even when the drug remains present (Hawkins et al., 2015).

Ke et al. (2024) demonstrated that *C. elegans* is a suitable model for investigating drug tolerance to amphetamine-like substances (Ke et al., 2024). Owing to its conserved neurotransmitter systems, quantitative behavioral outputs, and experimental accessibility, *C. elegans* has also been widely applied to dissect mechanisms underlying ethanol-induced functional tolerance. Several molecular pathways have been implicated in nematode tolerance phenotypes. Ethanol-induced acute tolerance involves conserved regulators such as the large-conductance calcium-activated potassium (BK) channel SLO-1, the neuropeptide receptor NPR-1, cholinergic signaling, lipid-related pathways, and c-Jun N-terminal kinase (JNK)-dependent stress signaling (Bhandari et al., 2024; Jee and Batsaikhan, 2024). In addition to ethanol, repeated amphetamine exposure can reduce amphetamine-induced swimming-induced paralysis, supporting the use of *C. elegans* for studying psychostimulant tolerance (Torres Valladares et al., 2020). These findings indicate that tolerance assays in *C. elegans* provide a tractable platform for analyzing drug-induced behavioral adaptation and for identifying conserved molecular regulators of repeated drug exposure (Bhandari et al., 2024; Ke et al., 2024).

### 3.4 Acute stimulus chemotaxis assay

**FIGURE 2:**
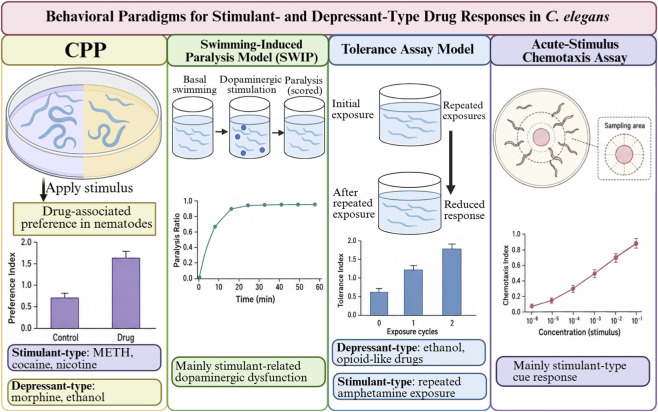
Behavioral paradigms for drug responses in *Caenorhabditis elegans*. The schematic summarizes conditioned place/cue preference, swimming-induced paralysis, tolerance assay, and acute-stimulus chemotaxis assay for assessing drug-associated preference, dopaminergic dysfunction, tolerance, and cue-guided responses.

The acute stimulus chemotaxis assay provides a rapid behavioral readout for stimulus-guided responses to drug-associated cues. By quantifying directional movement within a chemical gradient, this assay evaluates how sensory cues are translated into behavior (Bargmann et al., 1993). Evidence suggests that *C. elegans* exhibits non-random, stimulus-specific responses when encountering psychostimulants such as methamphetamine (METH), with higher concentrations inducing stronger attraction (Engleman et al., 2018). In addition to methamphetamine-induced chemotaxis, nicotine-related chemotactic responses can be modulated by nicotinic receptor antagonists such as mecamylamine or varenicline, and cocaine-associated conditioned cue preference has been linked to dopaminergic signaling (Sobkowiak et al., 2011; Sellings et al., 2013; Musselman et al., 2012). These findings indicate that chemotaxis-related paradigms can be used not only for a single psychostimulant but also as part of a broader behavioral framework for analyzing drug-associated cue responses. Together, these behavioral paradigms support the use of *C. elegans* for quantifying drug-associated preference, dopaminergic dysfunction, tolerance, and stimulus-guided behavioral responses (Figure 2).

## 4 Depressant- and stimulant-type drugs

This section is organized according to drug category. Opioid-, anesthetic-, and other depressant-type drugs are mainly related to neurotoxicity, tolerance, neuromuscular changes, GPCR signaling, and ion-channel regulation. Stimulant-type drugs are mainly related to dopamine signaling, monoamine transport, cholinergic receptor regulation, behavioral sensitization, and transcriptional or epigenetic changes. This organization helps compare different drugs while keeping their specific mechanisms clear.

### 4.1 Depressant-type and opioid/anesthetic-related drugs

#### 4.1.1 Opioid-induced behavioral changes and addiction-related phenotypes

Opioids are analgesic and highly addictive compounds that act primarily through opioid receptor-mediated GPCR signaling and can produce reward, tolerance, and withdrawal-related adaptations. Synthetic opioids, especially fentanyl and related analogs, and adulterants such as xylazine have become important concerns in the United States, Canada, and Mexico (Edinoff et al., 2024; Bradford et al., 2024; Bautista and Medina-Mora, 2024). Direct studies of these substances in *C. elegans* are still limited (Coleman et al., 2024). Nevertheless, the model may be useful for preliminary testing of opioid-like signaling, neuromuscular toxicity, and drug-interaction phenotypes, followed by validation in mammalian systems.

Through a complex interplay of monoaminergic and peptidergic signaling-including serotonergic and adrenergic components-*C. elegans* maintains an endogenous opioid-like system for nociceptive regulation (Beets et al., 2023). The NLP-24/NPR-17 signaling axis is central to this process, where the neuropeptide ligand NLP-24 modulates synaptic transmission via its cognate G protein-coupled receptor (GPCR), NPR-17 (Bhat et al., 2021). Available evidence suggests that morphine-sensitive NPR-17 signaling may influence dopaminergic modulation and addiction-related behavioral outputs in *C. elegans*. These behavioral changes are typically characterized by significant alterations in the organism’s chemotaxis and locomotion profiles (Cheong et al., 2015). Available evidence indicates that *C. elegans* can model certain opioid-induced behavioral adaptations, including preference and withdrawal-like responses, which depend on conserved neuromodulatory pathways (Ide et al., 2021). The effects of opiates in *C. elegans* are not limited to reward-related phenotypes. Opiates also modulate nociceptive behaviors through monoaminergic and peptidergic signaling pathways, suggesting that opioid-like signaling influences multiple neural circuits in the nematode (Mills et al., 2016).

#### 4.1.2 Opioid-related GPCR signaling pathways

Activation of opioid receptors triggers downstream G protein-mediated signaling pathways. These pathways are involved in regulating neuronal plasticity and circuit remodeling. In *C. elegans*, dopamine is synthesized in eight neurons in hermaphrodites (Wang et al., 2024). Harris et al. (2010) reported that NPR-17 is expressed in several head and tail neuron classes, including the AVG interneuron, ASI sensory neurons, PVP and PVQ interneurons, and the PQR sensory neuron (Harris et al., 2010). Although these neurons do not directly project to dopaminergic neurons, dopaminergic activity may be regulated indirectly through interneuron circuits receiving input from NPR-17-expressing neurons. Sensory and ion-channel pathways can substantially shape drug-evoked behavior in *C. elegans*. Transient receptor potential (TRP) family channels, as broadly acting signal integrators, have been reviewed as important regulators of diverse physiological and behavioral responses, providing candidate mechanisms for how environmental cues and internal states gate drug-related phenotypes (Xiao and Xu, 2009). Separately, opioid tolerance can be regulated by conserved trafficking mechanisms; for example, patched domain containing 1 (PTCHD1) has been identified as a key regulator of opioid tolerance through cholesterol-dependent effects on μ-opioid receptor trafficking and desensitization (Maza et al., 2022).

In opioid-like GPCR signaling, regulator of G protein signaling (RGS) proteins mainly function as negative-feedback regulators rather than upstream drivers of addiction-related behavior. After activation of NPR-17-mediated Gαi/o signaling, adenylyl cyclase activity is inhibited, leading to reduced cyclic adenosine monophosphate/protein kinase A (cAMP/PKA) signaling. RGS proteins accelerate GTP hydrolysis on activated Gαi/o and convert Gαi/o-GTP to Gαi/o-GDP, thereby shortening the duration and intensity of opioid-like GPCR signaling. Therefore, in *C. elegans* addiction-related assays, RGS proteins should be interpreted as intracellular feedback brakes that terminate NPR-17/Gαi/o signaling. Their specific function is to limit the duration and amplitude of opioid-like GPCR signaling rather than to directly initiate conditioned preference, withdrawal-like aversion, or tolerance-related adaptation (Stewart et al., 2015). Thus, decreased RGS activity may prolong opioid-like signaling, whereas increased RGS activity may dampen this pathway; however, direct genetic evidence for specific RGS isoforms in *C. elegans* opioid-addiction paradigms remains limited. These findings suggest that opioid-related responses in *C. elegans* are regulated by multiple interacting signaling pathways.

**FIGURE 3:**
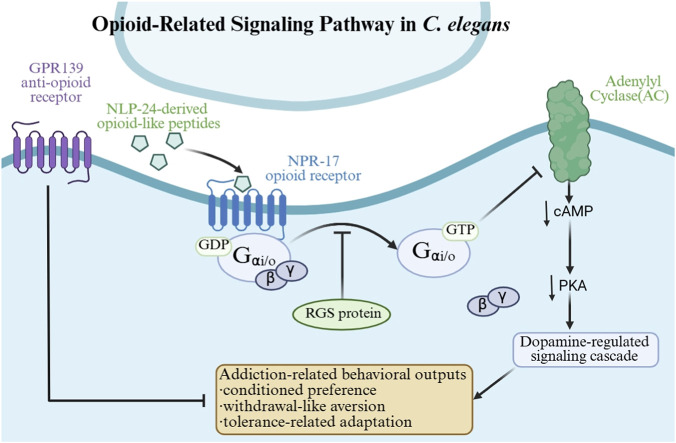
Depressant-type/opioid-related inhibitory signaling pathway in *Caenorhabditis elegans*. NLP-24-derived opioid-like peptides activate NPR-17/Gαi/o signaling, which inhibits AC-cAMP/PKA signaling and contributes to addiction-related behavioral outputs. RGS proteins terminate G-protein signaling, whereas GPR139 acts as an anti-opioid inhibitory branch.

Using forward genetics in *C. elegans*, Wang *et al.* identified a conserved orphan G protein-coupled receptor 139 (GPR139). This receptor exhibits anti-opioid activity. It is co-expressed with the μ-opioid receptor in opioid-sensitive neural circuits. GPR139 directly interacts with the μ-opioid receptor. It suppresses μ-opioid receptor signaling to G proteins (Wang et al., 2019). Current evidence does not indicate a direct NLP-24-GPR139 ligand-receptor relationship. Therefore, NLP-24 should be interpreted as acting through NPR-17, whereas GPR139 should be presented as a separate anti-opioid branch that suppresses μ-opioid-receptor-related G-protein signaling and downstream addiction-related behavioral outputs. In Figure 3, peptide-like symbols above NPR-17 indicate NLP-24-derived opioid-like peptides and should not be interpreted as evidence for a direct NLP-24-GPR139 pathway.

Deletion of *gpr-139* enhances opioid-mediated inhibition of neuronal activity and alters morphine-related analgesic, reward, and withdrawal responses (Dao et al., 2022). By regulating G protein signaling, opioid receptor activation may alter neuronal communication and may contribute to addiction-related phenotypes. These findings help clarify the molecular basis of opioid-related behavioral responses and suggest that NPR-17-, RGS-, and GPR139-associated pathways may represent candidate targets for further investigation (Figure 3).

#### 4.1.3 Propofol exposure, neurodevelopment, and cognitive function

Propofol is a short-acting intravenous anesthetic that potentiates inhibitory signaling and is widely used for induction and maintenance of anesthesia. However, increasing attention has been given to its potential neurotoxicity in the developing nervous system. Propofol exposure may interfere with brain development and may impair learning and cognitive functions (Zhang et al., 2024). In mammalian models, repeated propofol exposure can impair learning and memory and alter hippocampal synaptic plasticity (Fan et al., 2023). In *C. elegans*, early L1-stage propofol exposure reduces L4-stage learning ability and suppresses both non-associative and associative memory in a time- and dose-dependent manner (Zhao et al., 2023). These findings indicate that propofol broadly impairs multiple forms of memory and affects both memory formation and maintenance.

In addition to propofol, volatile anesthetics also induce persistent behavioral changes in *C. elegans*. Exposure to isoflurane during the L1 larval stage causes lifelong alterations in spontaneous behavior. It also induces changes in neural dynamics. These effects include reduced reversal frequency during crawling and abnormal activity of motor neural circuits (Wirak et al., 2020). Therefore, propofol and anesthetics may impair memory by disrupting neuronal growth and development. They interfere with synaptic function and neural circuit stability. In *C. elegans*, propofol exposure upregulates *rgs-3* and impairs learning and memory, whereas RNA interference (RNAi)-mediated knockdown of *rgs-3* alleviates these deficits (Zhao et al., 2023). Because EGL-4/protein kinase G (PKG) participates in activity-dependent nuclear signaling programs that regulate synaptogenesis, propofol-induced upregulation of *rgs-3* may impair synaptogenesis-related transcriptional programs by inhibiting nuclear accumulation of EGL-4 (Yee et al., 2024). These findings suggest that propofol-related cognitive impairment in *C. elegans* may involve RGS-3/EGL-4-dependent disruption of synaptic plasticity.

#### 4.1.4 Ketamine-induced behavioral and molecular alterations

Ketamine is a dissociative anesthetic and N-methyl-D-aspartate receptor antagonist with clinical anesthetic and antidepressant applications, but repeated or nonmedical exposure can produce neurobehavioral toxicity and dependence-related concerns. Studies using the *C. elegans* model revealed that ketamine specifically induces apical extracellular matrix (aECM) modifications in the vulva and cuticle. Ketamine-treated worms exhibit normal vulval opening accompanied by abnormal vulval eversion. This phenotype suggests alterations in the aECM factor network independent of the chondroitin sulfate pathway. Ketamine improves locomotor defects in cuticle collagen-deficient coiled mutants. RNA sequencing analysis showed that approximately 30% of cuticle collagen genes are upregulated in response to ketamine (Yücel, 2022).

In addition to extracellular matrix remodeling, ketamine disrupts intracellular calcium homeostasis and neuromuscular signaling in *C. elegans*. Genetic analyses demonstrate that mutants of *unc-68*, which encodes the ryanodine receptor (RyR) homolog, exhibit pronounced hypersensitivity to ketamine, characterized by ketamine-dependent convulsions followed by paralysis. Molecular characterization revealed that the *unc-68*/*kra-1*(kh30) allele carries a serine-to-asparagine substitution at a protein kinase C phosphorylation site in the *ryr-1* gene, whereas *unc-68*(e540) is a splice acceptor mutation that introduces a premature stop codon. Both mutations impair ryanodine receptor–mediated calcium release, thereby amplifying ketamine-induced physiological dysfunction and suggesting that calcium signaling may contribute to ketamine-induced physiological toxicity (Sakube et al., 1997). In *C. elegans*, the ryanodine receptor is encoded by the *unc-68* gene. This receptor contains conserved RyR domains. It participates in egg-laying and embryonic development. It functions as a calcium-induced calcium release channel during muscle contraction. These features provide a foundation for studying the roles of the ryanodine receptor in embryogenesis and calcium-mediated muscle contraction in nematodes (Gao et al., 2024). Although these findings mainly reflect ketamine-induced physiological and neuromuscular toxicity, they may also provide mechanistic clues for understanding how ketamine perturbs conserved neuronal and behavioral regulatory pathways.

#### 4.1.5 Alcohol-induced behavioral and molecular adaptations

Ethanol is a central nervous system depressant that affects multiple neurotransmitter systems and ion channels, producing intoxication, tolerance, and withdrawal-related behavioral changes. *C. elegans* provides a tractable model for quantifying ethanol-induced state changes across contexts. Ethanol can produce behavioral disinhibition in worms, particularly in water-associated states where certain behaviors are normally suppressed; dopamine signaling, including D1-like receptor pathways, contributes to these context-dependent effects (Topper et al., 2014). Behavioral inhibition occurs only when internal ethanol concentrations reach levels comparable to intoxicating blood alcohol concentrations in humans. Egg-laying in *C. elegans* is under strong neuromodulatory control, and acetylcholine provides an inhibitory influence on the egg-laying circuit. Genetic and cellular analyses have defined key components and sites of action underlying acetylcholine-mediated inhibition of egg-laying behavior (Bany et al., 2003). Wild-type worms exhibit concentration-dependent preference for 50%, 70%, and 95% ethanol. Acute ethanol preference is blocked by naltrexone. *npr-17* opioid-like receptor mutants do not display ethanol preference. Mutants with elevated *npr-17* expression show enhanced ethanol preference, which is attenuated by naltrexone. These findings suggest a possible role for NPR-17 signaling in ethanol reward-related behavior. *C. elegans* has emerged as a tractable system for dissecting ethanol-related behaviors and for identifying candidate pathways relevant to alcohol use disorders (Katner et al., 2019).

Ethanol exposure can alter sensory processing and behavioral plasticity in *C. elegans*. Recent work shows that ethanol modulates mechanosensory habituation, indicating that alcohol can reshape experience-dependent responses in defined circuits (Kokan et al., 2025). In parallel, conserved molecular determinants within BK potassium channels contribute to intoxication-related phenotypes, supporting ion-channel–level control over ethanol sensitivity (Davis et al., 2014). At the level of motivated behavior, neuropeptidergic pathways have been implicated in aversion-resistant or compulsive ethanol seeking in *C. elegans*, highlighting neuromodulatory control of persistent alcohol-directed behavior (Salim et al., 2022). Tolerance is a key feature of alcohol dependence. In *C. elegans*, natural variation in the neuropeptide receptor homolog *npr-1* modifies ethanol responses, providing mechanistic insight into genetic regulation of ethanol-related behaviors (Davies et al., 2004). In mammals, γ-aminobutyric acid (GABA) is the major inhibitory neurotransmitter in the central nervous system, and high-dose ethanol can enhance GABAergic signaling. In *C. elegans*, ethanol exposure downregulates genes involved in GABA signaling, including *unc-25*, *unc-47*, and *unc-49*, suggesting that GABAergic adaptation may contribute to ethanol-related tolerance (Davies et al., 2003). In mammalian alcohol models, neuropeptide Y (NPY) signaling can regulate GABAergic transmission in the central amygdala and influence ethanol-related behaviors and excitation–inhibition balance (Heilig, 2023). These findings provide cross-species context for understanding how neuropeptidergic and inhibitory signaling pathways may shape alcohol-related behavioral adaptation.

Transcriptomic analyses indicate that ethanol exposure elicits broad, time-dependent gene-expression responses in *C. elegans*, providing a systems-level view of alcohol-induced cellular programs (Sterken et al., 2021). Mechanistically, invertebrate models have been used to summarize synaptic adaptations underlying ethanol tolerance and neuroplasticity, offering conserved hypotheses for circuit-level remodeling with repeated exposure (Bhandari et al., 2024). In mammals, neuropeptide systems such as basolateral amygdala neuropeptide Y (NPY) have been linked to binge-drinking–related phenotypes and anxiety-like states, illustrating complementary neuropeptidergic control of alcohol-related behavior across species (Robinson et al., 2024). Ethanol-induced hypercontraction (EHC) in *C. elegans* depends on cholinergic signaling and is reduced in *cha-1* and *unc-17* mutants or by pretreatment with the nicotinic acetylcholine receptor antagonist mecamylamine. Wild-type worms develop tolerance to EHC, whereas the Na^+^/K^+^-ATPase mutant *eat-6*(eg200) fails to develop this tolerance (Hawkins et al., 2015). Cholinergic function is also regulated by long-chain polyunsaturated fatty acids. Mutants deficient in these fatty acids show reduced initial ethanol sensitivity and fail to develop acute tolerance (Jee et al., 2022). These findings indicate that cholinergic signaling, lipid metabolism, and Na^+^/K^+^-ATPase function contribute to ethanol-induced activation and tolerance in *C. elegans*, although the relevance of these mechanisms to vertebrates requires further investigation.

**FIGURE 4:**
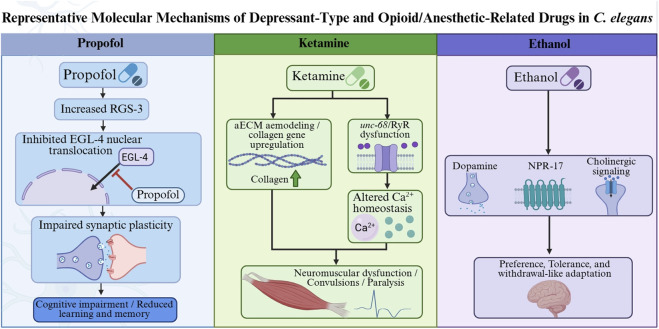
Representative molecular mechanisms of depressant-type and opioid/anesthetic-related drugs in *Caenorhabditis elegans*. Propofol increases RGS-3 and inhibits EGL-4 nuclear translocation; ketamine promotes aECM/collagen remodeling and disrupts UNC-68/RyR-mediated Ca^2+^ homeostasis; ethanol engages dopamine, NPR-17, and cholinergic signaling. These pathways converge on synaptic plasticity, neuromuscular dysfunction, preference, tolerance, and withdrawal-like adaptation.

Alcohol acts through multiple conserved neurotransmitter pathways in *C. elegans*, supporting the use of this model for mechanistic studies of alcohol-related behaviors (Zhu et al., 2014). The c-Jun N-terminal kinase (JNK) signaling pathway is a key regulator of cellular stress responses. It has been implicated in alcohol-related behavioral and stress-response phenotypes. JNK signaling mediates acute ethanol tolerance in *C. elegans* (Jee and Batsaikhan, 2024). Epigenetic studies using ethanol behavioral assays in *C. elegans* identified conserved components of the switch/sucrose non-fermentable (SWI/SNF) chromatin remodeling complex. These genes regulate ethanol-induced gene-expression programs and physical dependence to ethanol (Mathies et al., 2026). Twelve SWI/SNF-related genes were identified in worms. In humans, allelic variation in SWI/SNF genes is associated with alcohol dependence in genome-wide association studies. UNC-18 also modulates ethanol sensitivity in *C. elegans* (Graham et al., 2009). Ethanol activates a Gαs-cAMP-protein kinase A signaling pathway in IL2 neurons to stimulate locomotion, and heat shock protein HSP-16.48 and heat shock factor HSF-1 act as regulators of this excitatory response (Johnson et al., 2017). Small-molecule modulators of sigma-2 receptor/transmembrane protein 97 (σ2R/TMEM97) reduce alcohol withdrawal-induced behaviors, suggesting that σ2R/TMEM97 may represent a candidate target for further validation in alcohol use disorder research (Scott et al., 2018). These depressant-type and opioid/anesthetic-related mechanisms are summarized in Figure 4.

These studies can be divided into two practical groups. Opioid and ethanol studies mainly involve GPCR or neuropeptide signaling, ion channels, tolerance, and withdrawal-like behavior. Propofol and ketamine studies mainly involve learning defects, neuromuscular changes, calcium signaling, and extracellular-matrix remodeling.

### 4.2 Stimulant-type drugs

The following subsections focus on three recurring mechanisms across stimulant-type substances: altered dopamine handling, receptor-dependent behavioral sensitization or tolerance, and transcriptional or epigenetic adaptation.

#### 4.2.1 Methamphetamine-induced behavioral and molecular adaptations

METH is a highly addictive psychostimulant, and the genetic tractability of *C. elegans* makes this organism a useful model for studying METH-induced neurotoxicity and neuroadaptation. In addition to behavioral phenotypes, the nematode model allows mechanistic analysis of METH-induced cellular stress responses, dopaminergic dysfunction, and adaptive protective pathways. METH exposure alters foraging behavior in *C. elegans*. It changes food preference and disrupts feeding patterns (Katner et al., 2016). METH also reduces survival rates. It impairs reproductive capacity. In some cases, it causes cumulative damage to offspring. Most neurogenesis in *C. elegans* occurs during embryogenesis. METH exposure during this period is toxic to dopaminergic neurons. This toxicity is accompanied by reduced dopamine levels in adult worms. The *nsy-1* gene encodes a mitogen-activated protein kinase kinase kinase (MAPKKK), the *C. elegans* homolog of mammalian ASK1/MAP3K5, and functions in the conserved p38 MAPK innate immune pathway (Kim et al., 2002). The lethal effects of METH are significantly attenuated in *nsy-1* mutant worms (*nsy-1*[eg691]). NSY-1 functions upstream in the highly conserved p38 mitogen-activated protein kinase (MAPK) pathway. This pathway has been implicated in toxic responses to dopamine, methamphetamine, and 3,4-methylenedioxymethamphetamine (MDMA), suggesting that p38 MAPK signaling may contribute to monoaminergic neurotoxicity induced by amphetamine-type stimulants (Schreiber and McIntire, 2011).

At present, the relationship between METH-induced neurotoxicity and its reward-related or addictive properties remains incompletely understood. *C. elegans* provides a tractable model for investigating this relationship and may facilitate the identification of signaling pathways involved in METH-induced neuroadaptation and neurotoxicity. Overall, METH studies in *C. elegans* highlight mechanisms involving dopamine overload, reactive oxygen species (ROS) production, and DAF-16/forkhead box O (FOXO), cytochrome P450 (CYP), and p38 MAPK-associated adaptive protective responses.

#### 4.2.2 Cocaine-induced behavioral and molecular adaptations

Cocaine is a psychostimulant that blocks monoamine transporters and alters dopaminergic, serotonergic, and other monoaminergic signaling. *C. elegans* possesses a conserved monoaminergic nervous system, with dopaminergic signaling pathways showing strong molecular and functional similarity to those of vertebrates, including humans, supporting its utility as a model for investigating psychostimulant-induced behaviors and underlying molecular mechanisms (Muralidhara and Hardege, 2025). The dopamine transporter DAT-1 in *C. elegans* is sensitive to psychostimulants and provides a mechanistic entry point for examining how cocaine and related drugs alter locomotor and cue-associated phenotypes.

Ward et al. reported that high concentrations of cocaine reduce locomotor speed in *C. elegans*. This effect is mediated by the serotonergic system. Cocaine responses in *C. elegans* depend on serotonin signaling. Cocaine-induced behavioral changes are primarily mediated by the serotonin-gated chloride channel MOD-1 (Ward et al., 2009). Thus, cocaine mainly regulates locomotion through the serotonergic system in *C. elegans*. Musselman H. N. et al. used a Pavlovian chemosensory conditioning paradigm. Cocaine was paired with environmental cues such as specific food or salt stimuli. Cocaine at concentrations of 5–50 μM significantly enhanced preference for salt or food cues. This effect was abolished in dopamine synthesis–deficient mutants. These mutants included *cat-1* mutants with defective vesicular monoamine packaging and *cat-2* mutants lacking tyrosine hydroxylase activity. Exogenous dopamine supplementation restored cocaine-associated conditioned preference in these mutants (Musselman et al., 2012). Taken together, *C. elegans* provides a useful model system for investigating cocaine-induced reward-related and behavioral responses.

#### 4.2.3 Nicotine-induced behavioral and molecular adaptations

Nicotine is a cholinergic addictive compound that acts mainly through nicotinic acetylcholine receptors and produces dependence-related changes in receptor sensitivity and neural adaptation. Chronic nicotine exposure upregulates nicotinic acetylcholine receptors (nAChRs), a process that may contribute to nicotine dependence in humans and animal models. Jones A. K. and Rand J. B. reported that *C. elegans* expresses at least 29 distinct nAChR subunits (Jones and Sattelle, 2004). This diversity confers rich cholinergic pharmacology. Acetylcholine contributes to the regulation of reproductive behavior in *C. elegans*, and food sensing modulates this process through neuromodulatory disinhibition (Chen et al., 2025). Learning, memory, and reward-related behaviors in *C. elegans* are associated with specific nAChR subunits (Salim et al., 2024).

In *C. elegans*, chronic nicotine exposure alters locomotor behavior by sensitizing a mechanosensory circuit and thereby induces abnormal motor coupling (Liu Y. et al., 2025). Nicotine exposure induces muscle hypercontraction and triggers egg-laying in *C. elegans*. Prolonged nicotine exposure disrupts egg-laying control. This effect is regulated by the *unc-29* gene (Harrington et al., 2022). Chronic nicotine exposure leads to tolerance. Withdrawal induces locomotor incoordination. This tolerance is regulated by protein kinase C (PKC) (Rauthan et al., 2017). Nicotine-dependent behavioral responses in *C. elegans* require the TRP-family channels TRP-1 and TRP-2, supporting a role for TRP signaling in nicotine-related behaviors (Feng et al., 2006).

**FIGURE 5:**
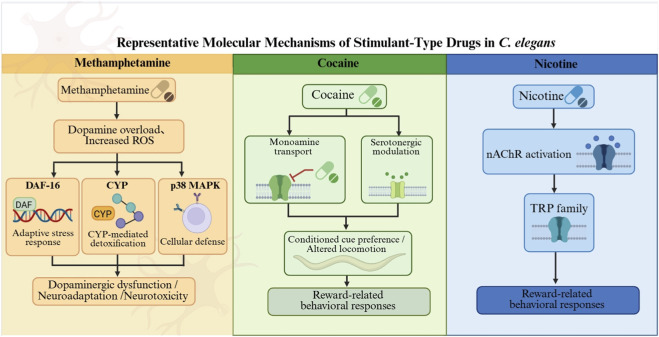
Representative molecular mechanisms of stimulant-type drugs in *Caenorhabditis elegans*. METH induces dopamine overload and ROS accumulation, involving DAF-16, CYP-mediated detoxification, and p38 MAPK-related defense. Cocaine alters monoamine transport and MOD-1-mediated serotonergic signaling, whereas nicotine activates nAChR and TRP-family signaling to regulate reward-related behavioral responses.

Nicotine-induced behavioral responses in *C. elegans* are concentration- and time-dependent, and nicotine chemotaxis can be inhibited by nicotinic receptor antagonists such as mecamylamine or by varenicline (Sobkowiak et al., 2011). Mutations in dopamine receptor genes (*dop-1*, *dop-2*) or nAChR subunit genes (*acr-5*, *acr-15*) reduce nicotine chemotaxis. Neuron-specific expression of *acr-15* restores nicotine chemotaxis in *acr-15* mutants. Muscle-specific expression does not rescue this phenotype (Sellings et al., 2013). Worms also display reward-like conditioned preference for nicotine-associated cues. This preference is abolished in *acr-5* mutants (Polli et al., 2015). Biogenic amine signaling provides an important framework for understanding drug-induced behavioral states in *C. elegans* (Chase and Koelle, 2007). Chronic nicotine exposure disrupts this regulatory balance by affecting *alg-1*-dependent and *miR-238*-related control of *acr-19* expression, thereby altering synaptic sensitivity. These findings suggest that microRNA-dependent regulation contributes to nicotine-induced neural and behavioral adaptations. Nicotine significantly alters the expression of approximately 40 microRNAs. These changes are dose-dependent. The microRNA system mediates a “regulated hormetic excitatory effect.” This effect involves microRNA-dependent regulation of the *fos-1* gene. It is maintained by epigenetic mechanisms (Hajiasgharzadeh et al., 2023). The mechanisms described for methamphetamine, cocaine, and nicotine are summarized in Figure 5.

## 5 Discussion

### 5.1 Limitations of the *Caenorhabditis elegans* model in addiction research

Despite these advantages, *C. elegans* models have important limitations. The nematode nervous system lacks the anatomical and functional complexity of mammalian mesolimbic reward circuitry, including cortical-striatal-limbic interactions that are central to compulsive drug seeking and relapse. In addition, drug responses in *C. elegans* may be influenced by nematode-specific pharmacokinetics, cuticle permeability, and differences in absorption, distribution, metabolism, and clearance. The absence of mammalian adaptive immune and endocrine complexity also limits direct extrapolation to human addiction. Therefore, findings from *C. elegans* should be interpreted as mechanistic and screening-level evidence that requires validation in mammalian models. To provide a clearer and more accessible overview of these points, the major advantages and limitations of *C. elegans* as a model organism for drug addiction research are summarized in Table 1.

**TABLE 1:** Major advantages and limitations of *Caenorhabditis elegans* (*C. elegans*) as a model for drug addiction research.

Advantages	Limitations
Simple nervous and visceral structures make *C. elegans* suitable for whole-organism analysis of drug responses	*C. elegans* lacks mammalian reward-circuit complexity and cannot fully model craving, compulsive drug seeking, or relapse
Convenient genetic manipulation and conserved human disease-associated homologs support mechanistic studies and target screening	Cuticle permeability and drug metabolism differ from mammals, limiting direct dose-response translation
Quantifiable behavioral phenotypes allow assessment of drug-associated preference, paralysis, tolerance, and chemotaxis	Simplified behavioral assays require validation in mammalian systems before clinical interpretation

Stimulant-type drugs in *C. elegans* models are mainly associated with dopaminergic or cholinergic activation, chemotaxis, swimming-induced paralysis, and oxidative stress-related adaptation, whereas depressant-type or opioid/anesthetic-related drugs are more closely associated with opioid-like GPCR signaling, ion-channel regulation, cognitive impairment, tolerance, and withdrawal-like adaptation. Therefore, the value of the nematode model lies in enabling dynamic analysis of drug-induced behavioral adaptation and conserved neurotransmitter regulation, rather than in serving as a simple substitute for the complex addiction phenotypes observed in mammals. Nematodes provide a useful model for studying selected mechanisms of drug addiction. Owing to their simple nervous system structure, a well-defined neural connectivity map, and highly conserved neurotransmitter signaling pathways such as dopamine, nematodes offer unique advantages for elucidating addiction-related behaviors and molecular mechanisms (Cook et al., 2019; Giunti et al., 2021). With continued drug exposure, nematodes may gradually develop addiction-related phenotypes, including conditioned preference for drug-paired cues, tolerance, and withdrawal-like responses, some of which depend on dopaminergic neurotransmission (Feng et al., 2006; Ide et al., 2021). Importantly, nematodes provide a useful system for elucidating conserved neurobiological mechanisms and for screening candidate intervention targets, although their translational relevance to human addiction remains limited by the preclinical nature of the current evidence (Engleman et al., 2016; Caldwell, 2023).

Dysfunction of the dopaminergic reward pathway is one of the important pathological features of drug addiction (Koob and Volkow, 2016). When compulsive drug use, craving, and relapse tendencies become prominent, addiction-related reward circuits are thought to have undergone persistent neuroadaptive remodeling. In nematode drug-exposure models, addiction-related behavioral phenotypes are associated with alterations in conserved neurotransmitter signaling, including dopaminergic and opioid-related pathways (Engleman et al., 2016; Ide and Ikeda, 2024). Nematodes can exhibit conditioned preference, drug-seeking-like behavior, tolerance, and withdrawal-like responses, supporting their utility for mechanistic studies of addiction-related neuroadaptation. These findings suggest that drug-induced neurotransmitter imbalance may be an early event in the formation of addiction-related behaviors and highlight the importance of remodeling conserved reward signaling networks during addiction progression.

Notably, conserved neurotransmitter signaling appears to be a key determinant of addiction-related behavioral phenotypes in nematodes. Previous studies have shown that stimulant-associated cue conditioning is closely linked to dopamine signaling (Musselman et al., 2012), whereas nicotine-motivated behavior is mediated by nicotinic acetylcholine receptors and also requires dopamine signaling (Salim et al., 2024). In addition, morphine can induce conditioned preference and withdrawal-like responses in nematodes, suggesting that opioid receptor-related pathways are also involved (Ide et al., 2021). Therefore, studying these conserved neurotransmitter- and reward-related signaling systems may provide useful mechanistic clues into the mechanisms of drug addiction and facilitate the screening of potential intervention targets using nematode models (Engleman et al., 2016). Although studies have shown that nematodes can exhibit addiction-related behavioral phenotypes such as conditioned preference, tolerance, and withdrawal, their application in drug addiction research still faces challenges, especially in improving the translational correspondence between these phenotypes and the core characteristics of human addiction. Because nematodes are amenable to behavioral screening and molecular interrogation, they may be particularly useful for elucidating addiction-related mechanisms and identifying candidate intervention targets (Kim et al., 2024). Furthermore, the establishment of experimental paradigms such as nicotine-motivated behavior and stimulant-related conditioned cue preference further supports nematodes as an experimental model for studying conserved addiction-related mechanisms (Musselman et al., 2012; Salim et al., 2024). Therefore, the value of the nematode model lies in enabling dynamic analysis of drug-induced behavioral adaptation and conserved neurotransmitter regulation, rather than in serving as a simple substitute for the complex addiction phenotypes observed in mammals.

Given the advantages and limitations of nematode models in drug addiction research, over-reliance on a single behavioral phenotype may limit a comprehensive understanding of addiction mechanisms and affect the translational interpretation of results. Therefore, relevant research strategies should focus on three core principles: First, conserved neurotransmitter pathways should be integrated with behavioral analyses to identify key drivers of early neuroadaptive changes during drug exposure. Second, more stable and standardized behavioral paradigms should be established to improve the reproducibility and comparability of phenotypes induced by different addictive substances. Third, genetic, pharmacological, and molecular approaches should be combined to screen potential intervention targets and enhance the value of nematode models in addiction mechanism research and early drug discovery.

## 6 Conclusion

This review summarizes the important role of *C. elegans* models in research on drug addiction-related behaviors and molecular adaptations. Existing evidence suggests that *C. elegans* can exhibit addiction-related behavioral phenotypes such as conditioned preference, tolerance, and withdrawal, and can be used to analyze the roles of dopamine and other conserved neurotransmitter pathways in drug-induced behavioral adaptation. However, despite their advantages in elucidating conserved mechanisms, *C. elegans* cannot fully mimic the complex neural circuits and clinical features of mammalian addiction. Given these advantages and limitations, *C. elegans* models may serve as useful tools for elucidating conserved mechanisms of drug-induced behavioral adaptation and as preliminary platforms for screening candidate intervention targets, while translational interpretation should remain dependent on validation in mammalian systems. Future studies that improve the standardization of behavioral paradigms and integrate genetic, pharmacological, and molecular approaches may strengthen the value of *C. elegans* for elucidating addiction mechanisms and screening early intervention strategies.
